# A Skewed Loss Function for Correcting Predictive Bias in Brain Age Prediction

**DOI:** 10.1109/TMI.2022.3231730

**Published:** 2023-06-01

**Authors:** Hanzhi Wang, Matthias S. Treder, David Marshall, Derek K. Jones, Yuhua Li

**Affiliations:** School of Computer Science and Informatics, Cardiff University, CF10 3AT Cardiff, U.K; Cardiff University Brain Research Imaging Centre, Cardiff University, CF24 4HQ Cardiff, U.K; School of Computer Science and Informatics, Cardiff University, CF10 3AT Cardiff, U.K

**Keywords:** Brain age delta, deep learning, neuroimaging, skewed loss function, regression bias correction

## Abstract

In neuroimaging, the difference between predicted brain age and chronological age, known as *brain age delta*, has shown its potential as a biomarker related to various pathological phenotypes. There is a frequently observed bias when estimating brain age delta using regression models. This bias manifests as an overestimation of brain age for young participants and an underestimation of brain age for older participants. Therefore, the brain age delta is negatively correlated with chronological age, which can be problematic when evaluating relationships between brain age delta and other age-associated variables. This paper proposes a novel bias correction method for regression models by introducing a skewed loss function to replace the normal symmetric loss function. The regression model then behaves differently depending on whether it makes overestimations or underestimations. Our approach works with any type of MR image and no specific preprocessing is required, as long as the image is sensitive to age-related changes. The proposed approach has been validated using three classic deep learning models, namely ResNet, VGG, and GoogleNet on publicly available neuroimaging aging datasets. It shows flexibility across different model architectures and different choices of hyperparameters. The corrected brain age delta from our approach then has no linear relationship with chronological age and achieves higher predictive accuracy than a commonly-used two-stage approach.

## I Introduction

DUE to the increasing risk of age-related brain diseases, brain age prediction has attracted a growing interest in recent years. It can be formulated as building a regression model that takes structural brain magnetic resonance imaging (MRI) data from healthy individuals as input and uses chronological ages, i.e., the age from birth, as output. Aging can cause marked changes in the brain-aging trajectory and deviations from the healthy brain-aging trajectories can indicate the risk of age-related brain diseases [1]. To measure this deviation, a metric known as *brain age delta*, defined as the difference between an individual’s estimated brain age and chronological age has been proposed [2].

In predicting brain age, an age-related bias has been frequently observed [2], [3], [4], [5], [6], [7], [8], [9]. The predicted brain age tends to become older than the actual chronological age for young participants and younger for older participants. A useful quantification of this bias is the correlation between chronological age and brain age delta, also known as the *age delta correlation* (ADC). In this way, the bias manifests as a negative ADC value.

A nonzero ADC significantly weakens the validity of the brain age delta as a biomarker. Spurious relationships could then naturally arise between brain age delta and other variables of interest if these variables are also correlated with age [5]. It may also raise problems for subsequent experiments. For example, when investigating if the brain age delta differs across groups with different degrees of cognitive impairment, the differences in the brain age delta between groups may simply due to the group differences in chronological age distributions [10]. In that way, the apparent relationship between the brain age delta and variables of interest might be more driven by age and not the brain age delta [6].

Different approaches have been developed to mitigate the dependence of the brain age delta on age [4], [5], [6], [11], [12], [13]. Most of them can be summarized as a two-stage approach as they involve firstly training a brain age estimation model and applying a bias correction afterward on the model predictions. However, this explicit correction approach is a post-hoc correction of the model predictions (using a biased model) which can lead to sub-optimal results.

**Fig. 1:**
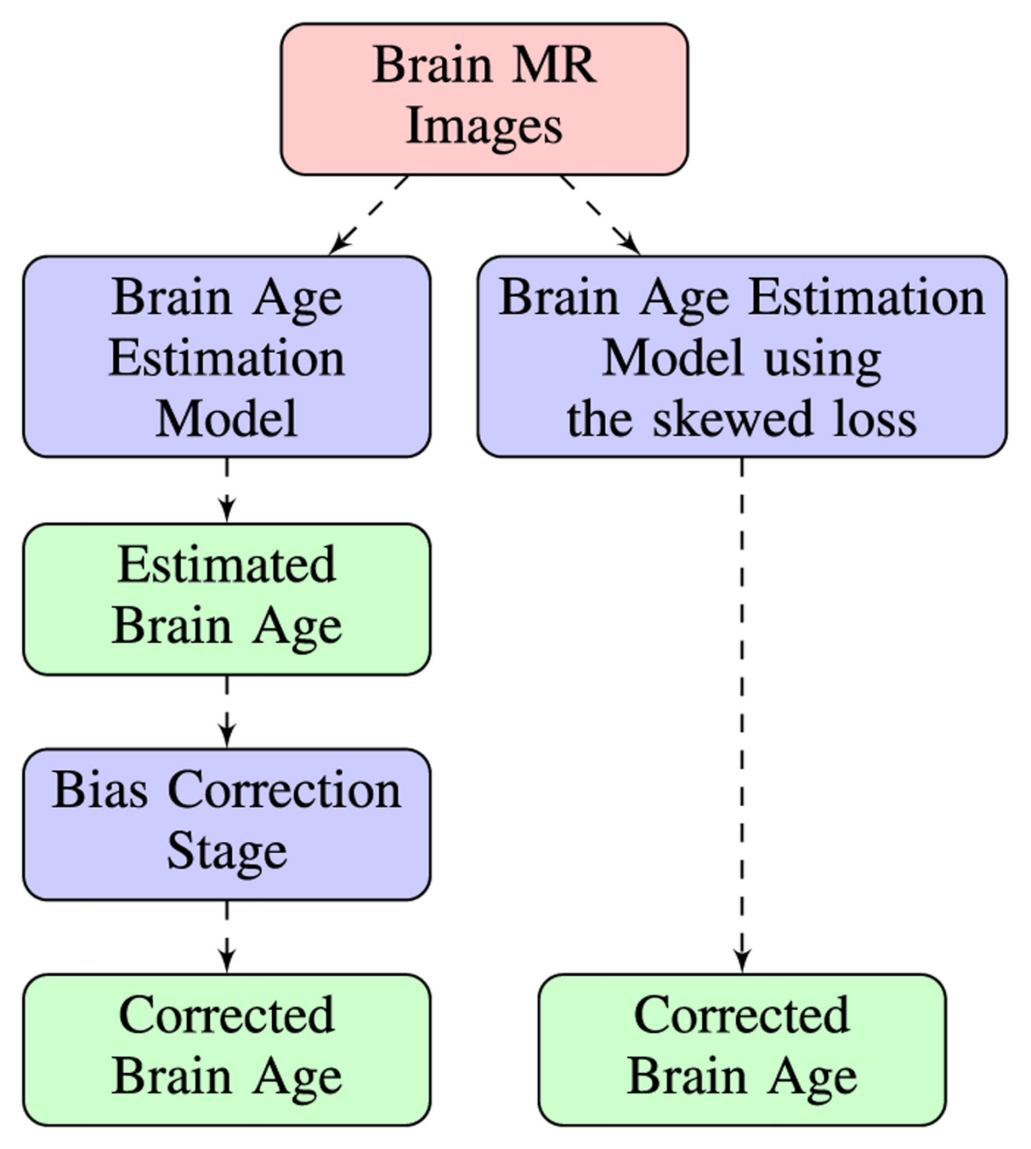
The overall workflow for the Brain Age Prediction problem. The left route represents the widely adopted two-stage approach and the right one represents our proposed approach. Each “Corrected Brain Age” block represents the final estimation of brain age for each approach and the corrected brain age should have no linear relationship with chronological age. Two estimates of corrected brain age are then compared with chronological age respectively to see which one achieves a lower mean absolute error.

In this paper, we propose a novel approach to correct this bias. Compared with existing correction approaches, our approach only consists of a single training stage without the need to apply a bias correction stage. Model predictions at the end of the training process are unbiased. The overall workflow of the two-stage approach and our proposed approach is illustrated in Figure 1. Also, we would like to stress that our approach only modifies the loss function and can be combined with any existing differentiable models and any model architectures (e.g. ResNet [14], VGG-19 [15]). The implementations are available in the GitHub repository.^1^

Our main contributions in this paper are:

1) We propose a novel approach that corrects predictive bias directly in the model training stage. Compared with the commonly used two-stage approach, our method does not require an explicit bias correction stage. The proposed approach acts as an alternative correction method to the two-stage approach, whereas it achieves significantly better accuracy. To the best of our knowledge, this is the first such approach for deep learning models.
2) We develop a training strategy to find the optimal parameters for our method, which has been proven to be robust to different datasets and model architectures.

The paper is organized as follows. Section II reviews the recent developments in brain age prediction and existing approaches to tackle the nonzero ADC problem. Section III proposes the skewed loss function to solve the observed bias. Section IV introduces a robust training strategy for the skewed loss to make the model performances consistent. Section V explains the settings of the experiments, including datasets and models. Section VI demonstrates experiment results and model comparisons. Section VII generalizes the skewed loss to other areas using the apparent age prediction problem. Section VIII summarizes the overall approach to conclude this paper.

## II Related Work

### A Brain Age Prediction Models

There has been a variety of studies that apply different machine learning techniques, such as ridge regression [7], support vector regression [16] and Gaussian process regression [2] to estimate brain age. Different convolutional neural network (CNN) architectures have also been applied to this task, such as VGG architecture [2], ResNet architecture [17], Inception architecture [18] and fully convolutional network architecture [8]. Despite adopting classical CNN architectures, these models have already shown superior predictive performances in brain age prediction.

### B Bias in Brain Age Delta

A more fundamental question in brain age prediction and the starting point of this paper is to investigate whether the brain age delta is an unbiased estimator. Several studies [2], [3], [4], [5], [6], [7], [8], [9] have observed that brain age delta is dependent on chronological age, which can be problematic in subsequent analysis. The observed bias is also known as regression dilution or regression attenuation, which could be found in many areas [19]. For example, in some epidemiology studies, it could behave as an underestimate of the association between the risk factor and the disease, such as blood pressure and stroke [20], [21], [22]. For brain age prediction, different explanations of the cause of this predictive bias have also been proposed. Liang et al. [7] found that a negative ADC value consistently arises in a range of aging datasets regardless of the regression models being used. Le et al. [5] proved mathematically that this bias is inevitable for regression models and hence not limited to aging datasets. Smith et al. [6] observed that a penalized regression model and a non-Gaussian distribution of the participants’ chronological age could cause the model to make predictions toward the mean age as well.

### C Bias Correction Approaches

In the literature, a two-stage approach has been proposed and widely adopted to correct the bias [4], [5], [6], [7], [11], [12]. It introduces a second-stage correction to correct the predictions from the first stage, i.e., the brain age estimation model and the resultant corrected brain age delta will then have no linear relationship with chronological age [12]. The two-stage approach can be summarized below:

1) brain age prediction: Train a regression model *f* to predict chronological age (Y) given brain MR images (X). The uncorrected brain age delta is then defined as
  (1) δ=f(X)−Y
2) bias correction: Remove the dependence of uncorrected brain age delta *δ* on the chronological age (Y).

Two different approaches have been proposed in the bias correction stage:

- Approach 1 [5], [6], [7], [11]:
  1) Fit a linear regression between uncorrected brain age delta *δ* and chronological age Y
    (2) $$\delta \;=\;\beta_{1}\times \;Y\;+\;\beta_{0}$$
  2) The corrected predicted age is defined as
    (3) $$f{\left(X\right)}_{corrected}=\;f(X)\;-\;(\beta_{1}\;\times \;Y\;+\;\beta_{0})$$
  3) The corrected brain age delta is then defined as
    (4) $$\delta_{corrected}=f{\left(X\right)}_{corrected}-Y$$
- Approach 2 [4], [12]:
  1) Fit a linear regression between predicted age *f (X)* and chronological age Y
    (5) $$f(X)=\;\;\beta_{1}\times \;Y\;+\;\beta_{0}$$
  2) The corrected predicted age is defined as
    (6) $$f{\left(X\right)}_{corrected}\;=\;(f(X)\;-\beta_{0})/\beta_{1}$$
  3) The corrected brain age delta is then defined as
    (7) $$\delta_{corrected}=f{\left(X\right)}_{corrected}-Y$$

Regarding the first approach, several studies [10], [13] have argued that this approach essentially corrects the target label (Y) and the model prediction is still biased. Moreover, in a machine learning framework, the target value (Y) from the test set should remain unknown when the correction approach is applied. Otherwise, one can always make corrections toward the target value (Y) to achieve a significantly lower error. The first approach uses the chronological age (target value) to make corrections in (3), which violates the principles of this predictive framework. Therefore, we will not include the first approach in the rest of this paper.

To apply the second approach in the predictive framework, the linear regression (5) is performed on the validation set and the resultant parameters (*β*_0_, *β*_1_) are assumed to be generalized to the test set. In that way, the predictions from the test set can be corrected using (6). In the rest of this paper, we refer to the second approach [4], [12] as the default two-stage approach and use it as the contrast in Section VI.

Although the two-stage approach has been proven to remove the age-related impact from brain age delta effectively in practice [4], [8], [12], it does not take ADC directly into account. Instead of correcting the predictions of a biased model, Treder et al. [13] proposed to fuse both stages by integrating a correlation constraint into the model training stage. This results in a regression model that is unbiased to start with and hence does not require post-hoc correction. However, the authors use an analytical solution for ridge regression and kernel ridge regression which does not extend to deep learning. To sum up, we propose a method that also solves the predictive bias at the model-building stage for any deep learning models. The method extends to any type of model that can be trained with a symmetric loss function (e.g. linear regression, Support Vector Regression).

## III Methodology

### A Skewed Loss Function

In this section, we introduce our approach for bias correction using the skewed loss functions. We start by observing that regression models are trained by minimizing a loss function. Commonly used loss functions include

(8) $$\begin{array}{l} meanabsoluteerror\;:\;{ℒ}_{mae}(y,\;\hat{y})\;=\;|y\;-\;\hat{y}|\\ meansquarederror\;:\;{ℒ}_{mse}(y,\;\hat{y})\;=\;|y\;-\;\hat{y}{|}^{2} \end{array}$$

where *y*, $\hat{y}$ represent the target label and predicted value.

Regression loss functions are typically symmetric, i.e., overpredictions are penalized as much as underpredictions. Therefore, one possible approach to counteract bias is to skew the functions and penalize overpredictions more than under-predictions for low values of *y* and vice versa. We denote this approach as a *skewed loss function* to reflect this characteristic. To this end, let us define a step function *s* : ℝ → ℝ as

(9) $$s(x)\;=\;\lambda_{0}\;1_{\mathbb{R}}{\;}_{<0}(x)\;+\;\lambda_{1}1_{{{\;}_{\mathbb{R}}}_{\geq 0}}(x)$$

where 𝟙*_U_ (x)* is the indicator function (1 if *x ∈ U* and 0 otherwise) and *λ*_0_ and *λ*_1_ are the heights of the steps. This implies that *s (x)* = *λ≥ 0.*

Multiplying the step function with the original loss function, we obtain the skewed loss functions

(10) $$\tilde{ℒ}(y,\;\hat{y})\;=\;ℒ(y,\;\hat{y})\;s(y\;-\;\hat{y})$$

We can further simplify *s (x)* to have only one hyperparameter *λ* that controls the amount of skew by setting $\lambda_{0}\;:=\exp(-\lambda ),\;\lambda_{1}\;:=\;\lambda_{0}^{-1}=\;\exp(\lambda ).$ Then *s (x)* simplifies to

(11) s(x)=exp(sgn(x)λ)

where sgn : ℝ → {-1, 1} is the sign function and *λ* controls the skew.

This simplification (11) reduces flexibility by imposing an “inverse symmetry” constraint on *λ*_0_ and *λ*_1_ in (9), whereas it also reduces model complexity with only one parameter controlling the behavior of the skewed loss.

To make the skewed loss function (10) compatible with the brain age prediction, two more adjustments are needed:

1) Skewed loss should behave differently in different age ranges. For young participants, it should assign more penalties to overpredictions than underpredictions. For elderly participants, it should assign more penalties to underpredictions than overpredictions.
2) The bias is more significant for participants with age closer to either end of the data range than those with ages closer to the mean age (of the training dataset) [12]. Participants with age closer to either end of the data range need larger levels of skew.

Therefore, we can further modify *s(x)* by setting *λ* as a function of chronological age (*y*). The range of *λ* is then constrained within [-*λ_max_*, +*λ_max_*] where *λ_max_* is a positive hyperparameter:

(12) s(x,y)=exp(sgn(x)λ(y))

Based on (12), a general guide to define *λ(y)* is that a smaller *y* should have a negative *λ* while a larger *y* should have a positive *λ*. We propose two approaches to define *λ(y)* using linear functions for simplicity.

#### • Approach 1

*λ(y)* can be defined as a linear function of *y*.

(13) $$\lambda (y)\;=\;g(y)\;\times \;\lambda_{max}\;+\;(1-g(y))\;\times \;(-\lambda_{max})$$

(14) $$g(y)\;=\;(y\;-\;y_{min})/(y_{max}\;-\;y_{min})$$

where *y_min_*, *y_max_* represent the minimum and maximum age of the dataset.

#### • Approach 2

*λ(y)* can be defined as a piecewise linear function of *y* by setting the median value of *y* in the dataset as a midpoint.

(15) $$\lambda (y)\;=\;\{\begin{array}{c} (1-g(y))\;\times \;\;(-\lambda_{max})\;if\;y\;\leq \;y_{med}\\ g(y)\;\times \;\lambda_{max}\;\;\;\;\text{otherwise} \end{array}$$

(16) $$g(y)\;=\;\{\begin{array}{c} (y\;-\;y_{min})/\;(y_{med}\;-\;y_{min})\;\;\;if\;y\;\leq \;y_{med}\;\\ (y\;-\;y_{med})/\;(y_{max}\;-\;y_{med})\;\;\text{otherwise} \end{array}$$

where *y_min_*, *y_max_*, *ymed* represent the minimum, maximum, and median age of the dataset.

The difference between these two approaches of defining *λ(y)* is that the first one assigns the middle value of *y* (in the training set) to have a zero *λ* value, whereas the second one uses the median value. For datasets with highly skewed age distributions, the second approach results in a roughly equal number of participants having positive *λ* values and negative *λ* values, which improves stability in practice.

By combining (10), (12), (15), and (16), we formally propose the skewed loss function for brain age prediction

(17) $$\tilde{ℒ}(y,\;\hat{y})\;=\;ℒ(y,\;\hat{y})\;s(y\;-\;\hat{y},\;y)$$

(18) $$s(y-\hat{y},\;y)\;=\;\exp(\mathrm{sgn}(y\;-\;\hat{y)\lambda }(y))$$

where *λ(y)* is defined using (15) and (16).

**Fig. 2:**
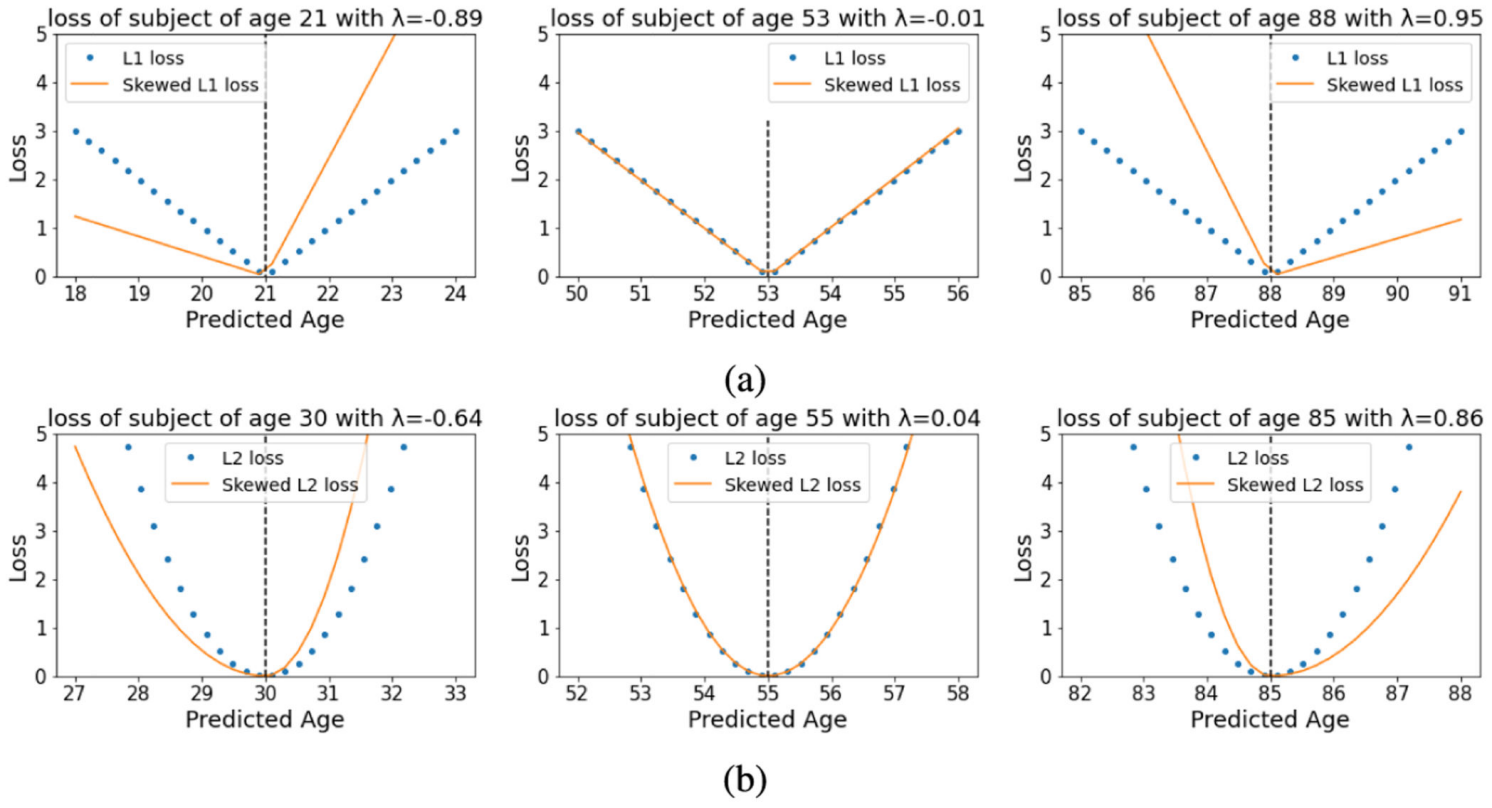
L1 and L2 skewed loss function illustration. For each plot, the dotted and solid lines represent the normal symmetric loss function and the skewed loss function respectively. The horizontal axis means possible model predictions and the vertical axis means the corresponding loss term for each prediction. For each plot, from left to right, the skewed loss behaves differently in different age ranges and different ages are assigned with different levels of skew using (15) and (16). *λ_max_* is set to 1 in both plots.

As an example, L1 and L2 skewed loss functions are illustrated in Figure 2.

### B Effect of the Skewed Loss

The idea of employing a skewed loss function is to assign different losses depending on whether the model makes overestimations or underestimations. Because the predictive bias manifests as an overestimation for younger individuals, we assign more penalties when the model overestimates ages for young participants to push the model to make fewer overestimations. This idea is reversed on elderly individuals and we then penalize the model more when it makes underestimations.

**Fig. 3:**
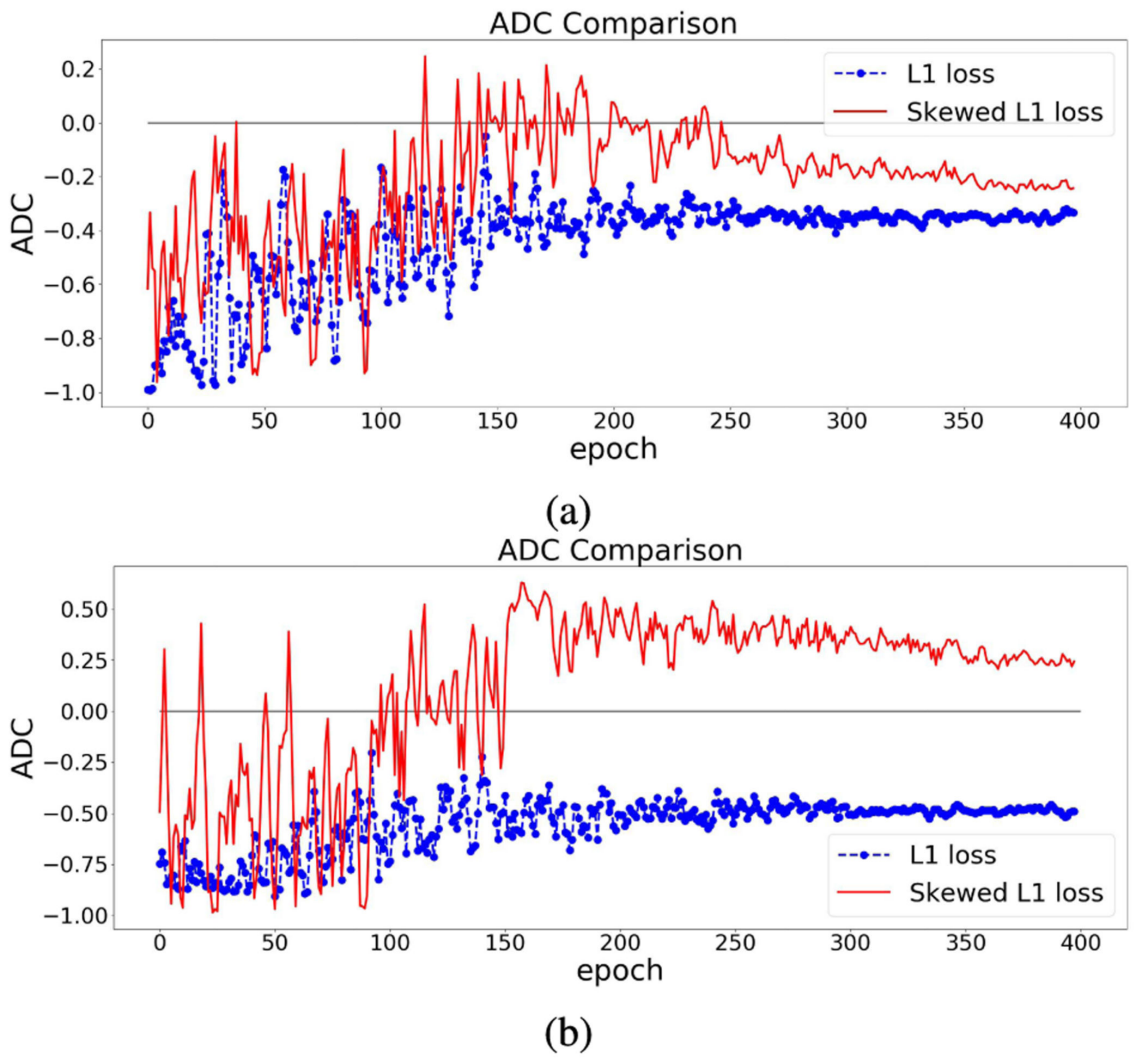
ADC Comparisons between L1 and skewed L1 loss. The model is trained twice using L1 and skewed L1 loss respectively. For each plot, the dashed and solid lines represent the changes of ADC (measured in Pearson’s r) on the validation set in the training process using L1 and skewed L1 loss respectively. *λ***max** is set as 1 in (a) and 2 in (b).

The effect of using the skewed loss in practice is illustrated in Figure 3. We can observe that models using symmetric L1 loss have negative ADC values at the end of the training, which is in line with previous studies, whereas models using skewed L1 loss end up with larger ADC values. Figure 3 shows that by applying the skewed loss functions, the model tends to make fewer overestimations for young individuals and fewer underestimations for elderly individuals so that the effect of a negative ADC is reduced.

## IV Dynamic Lambda Training Strategy

### A The Necessity of Dynamic Lambda Strategy

From Section III-A, *λ_max_* is a hyperparameter in the skewed loss which controls the skew of the loss function. From Figure 3, we can observe that setting *λ_max_* as 1 results in a small negative ADC while setting *λ_max_* as 2 ends up with a small positive ADC. That indicates there should be an optimal value between 1 and 2 for *λ_max_* so that the ADC can approach zero at the end of the training process.

However, due to the randomness of the network training process, different datasets used for training, and different model architectures being used, it is not realistic to foresee the optimal *λ_max_* that can push the ADC to zero before the training. To solve this problem, we propose a dynamic lambda training strategy to tune *λ_max_* in the training process to find the optimal value that is suitable for the current model. By dynamically changing *λ_max_* in the training process, we can enable the model predictions to have (near) zero ADC at the end of training.

### B Implementations of Dynamic Lambda Strategy

The dynamic lambda strategy is formally presented in Algorithm 1. Some notations in Algorithm 1 are defined in Table I.

It should be noted that hyperparameters in Table I are insensitive to different values and the “Range” column in Table I aims to give a general guide on how to set them.

In Table I, hyperparameters *α* and *β* separate the whole training process into three stages, all of which should have a similar number of epochs to ensure the model is properly trained in all stages. Hyperparameter *ε* determines how frequently we update *λ_max_*. A too-small value of *ε* may prevent the model from training properly toward the newly updated loss function, whereas a too-large value may prevent the model from finding the adequate value of *λ_max_* due to fewer updates. Hyperparameter *θ* acts as a positive threshold determining whether to adjust *λ_max_* according to the current ADC value. We will change *λ_max_* in the second stage only when the ADC falls outside of [-*θ, θ*]. Hyperparameter *η* determines how to change *λ_max_* in each update in the second stage.

### C Explanations forDynamic Lambda Strategy

Algorithm 1 divides the whole training process into three stages:

1) Stage-1: The aim is to pretrain the model with a fixed *λ_max_* to achieve relatively high accuracy before any modifications are made. This is done because due to the random initialization of the weights, ADC estimates in the first few epochs may be uninformative.
  Algorithm 1 **Dynamic Lambda Training Strategy**
  **Init**: *λ_max_ ←* 1; skewed loss ${ℒ}_{s}(\lambda_{max})$
  **for** *i* ← 1 **to** *γ* **do**
  **if** *i < α* **then** // stage-1
  Train the network using ${ℒ}_{s}(\lambda_{max})$
  **else if** *i < β* **then** // stage-2
  Train the network using ${ℒ}_{s}(\lambda_{max})$
  **while** *(i* – *β)* mod *ε* = 0 **do**
  Calculate the current *ADC* on the validation set
  Save *λ_max_* to M and *ADC* to N
  **if** *ADC > θ* **then**
  *λ_max_* ← *λ_max_/η*
  **else if** *ADC < – θ* **then**
  *λ_max_ ← λ_max_* × *η*
  **end**
  Reinitialize ${ℒ}_{s}(\lambda_{max})$ with updated *λ_max_*
  **end**
  **else** // stage-3
  Train the network using ${ℒ}_{s}(\lambda_{max})$
  **while** *(i – β)* mod *ĉ =* 0 **do**
  Calculate the current *ADC* on the validation set
  Save *λ_max_* to M and *ADC* to N
  Fit a linear regression *F* between M and N
  Select *λ_max_* resulting in a zero *ADC* using *F* as the optimal *λ_max_*
  Reinitialize ${ℒ}_{s}(\lambda_{max})$ with optimal *λ_max_*
  **end**
  **end**
  **end**
2) Stage-2: From this stage, we are trying to find the optimal *λ_max_* that results in a zero ADC on the validation set. It is generally assumed that the validation and test set should be derived from similar distributions, so we assume a zero ADC on the validation set should be generalizable to the test set. In this stage, we apply a heuristic method to try different *λ_max_* values to make ADC approach toward zero. We also save *λ_max_* and the corresponding ADC for stage-3.
3) Stage-3: Using the previously saved *λ_max_* and ADC pairs from stage-2, we can fit a linear regression model to find the optimal *λ_max_* that results in a zero ADC and set it as the updated *λ_max_*. This method is repeated multiple times until the end of training so that *λ_max_* is tuned iteratively. We use linear regression for its simplicity and it has been proven to be effective to find the optimal *λ_max_* in practice.

There is a slight increase in training time when using the skewed loss compared with using a symmetric loss function because of the training of linear models in stage-3. However, it is usually negligible compared with training a network.

Throughout the training process, our approach tries to control ADC explicitly by iteratively tuning *λ_max_*. Therefore, by measuring ADC with different types of correlation, our approach can find the optimal *λ_max_* resulting in the specified correlation approaching zero, which is not feasible for the two-stage approach.

**Fig. 4:**
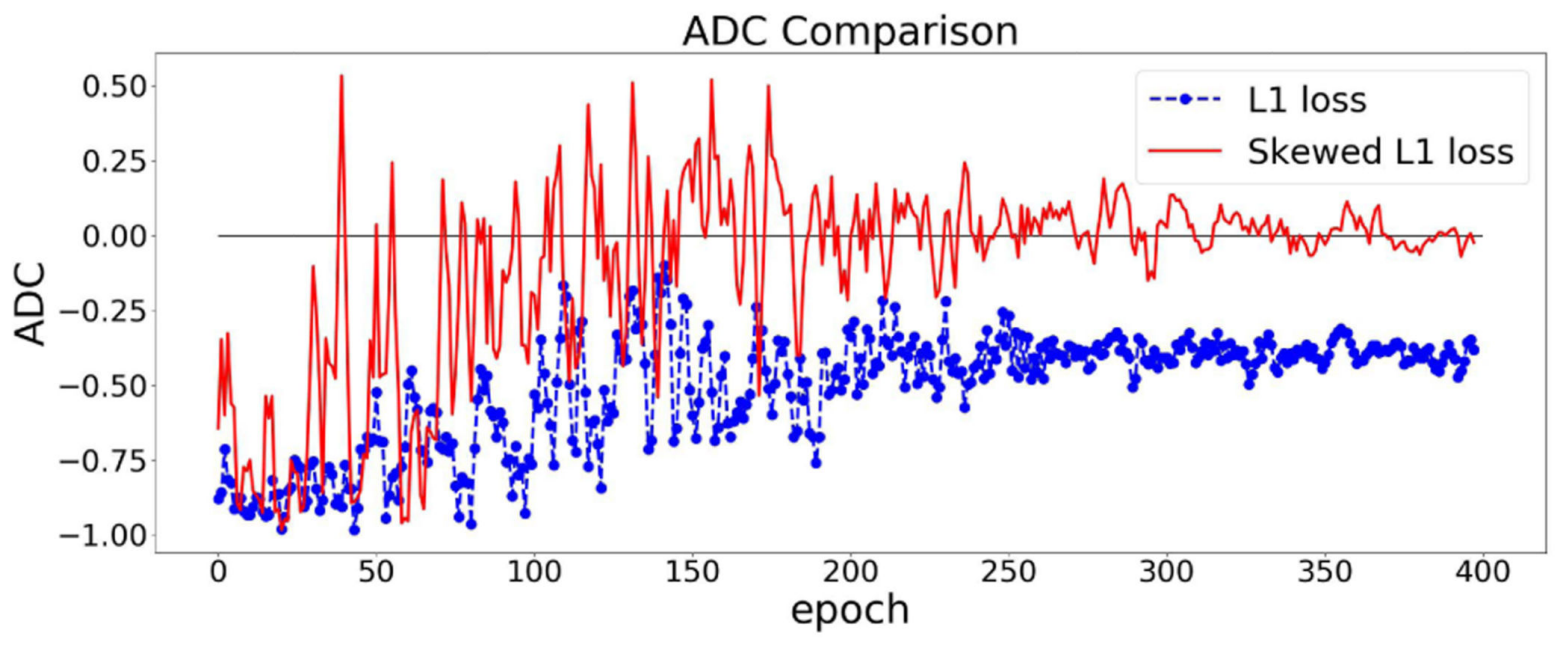
ADC Comparisons between L1 and skewed L1 loss. Dynamic lambda strategy is applied for the skewed loss. The model is trained twice using L1 loss and skewed L1 loss. The dashed and solid lines represent the changes of ADC (measured in Pearson’s r) on the validation set in the training process using L1 and skewed L1 loss respectively.

The effect of using the dynamic lambda strategy is illustrated in Figure 4. In Figure 4, we train our model twice using symmetric L1 loss and skewed L1 loss with dynamic lambda strategy. It can be easily observed that at the end of the training, the model using skewed loss ends up with a (near) zero ADC on the validation set.

## V Experiments

To empirically investigate our approach, we conducted experiments using two publicly available aging datasets and several classical neural network architectures. The models were trained using the normal symmetric loss and the skewed loss respectively. The two-stage approach was applied to models using the normal symmetric loss as the contrast.

### A Datasets

**Fig. 5:**
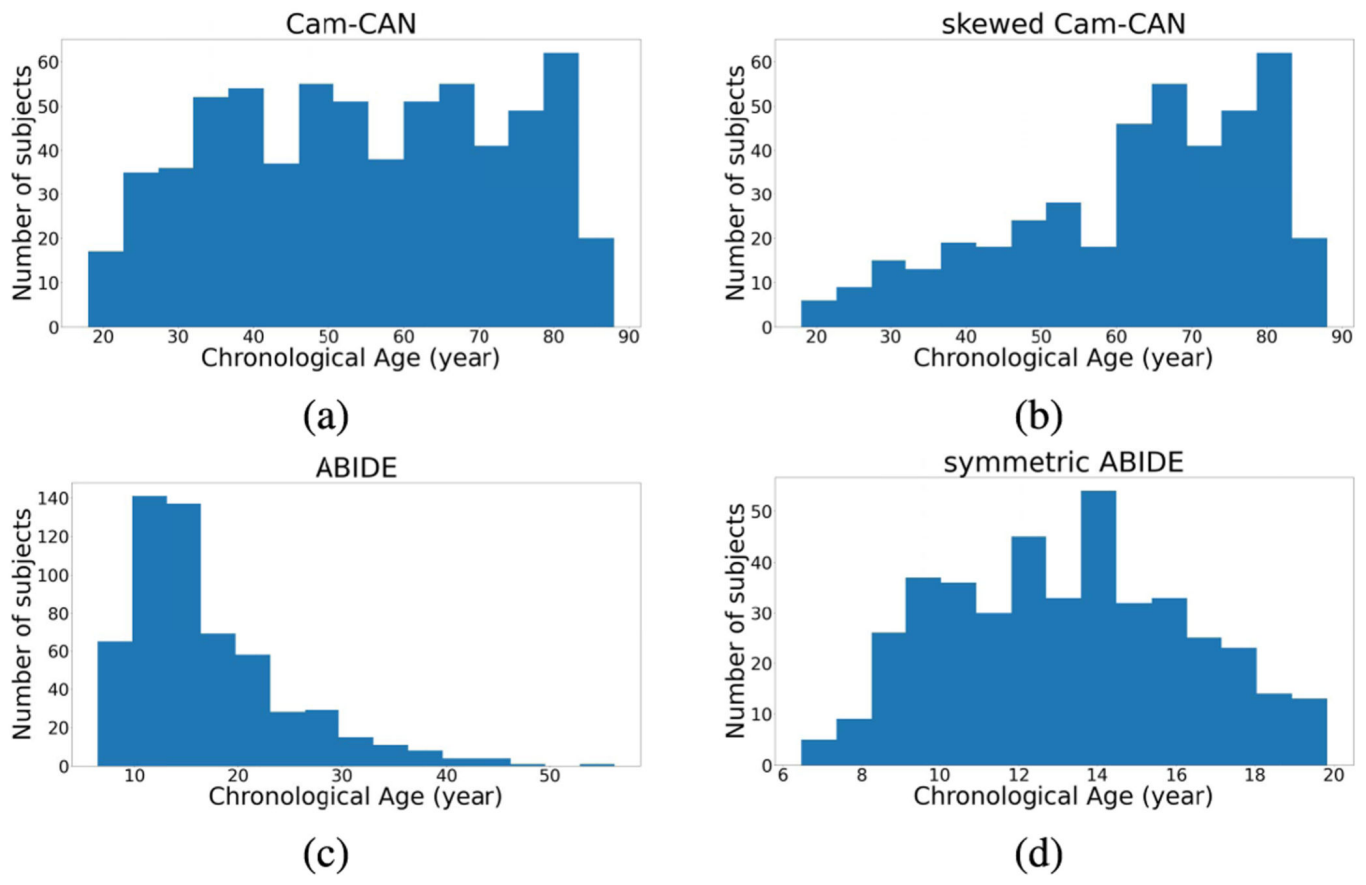
Age distribution for the Cam-CAN and ABIDE datasets and their skewed versions.

To validate the robustness of the skewed loss, we selected two public neuroimaging datasets, Cam-CAN [23], [24] and ABIDE [25], with preprocessed 3D structural T1-weighted MR brain images. The dataset descriptions and the specific preprocessing pipeline for each dataset could be found at the Cam-CAN^2^ and ABIDE^3^ website. The data from the Cam-CAN are preprocessed using the Automatic Analysis pipeline [26] and we selected the gray-matter density maps from it. The data from the ABIDE derives from cortical thickness measures using ANTs pipeline [27] and we selected the 3D volume containing voxel-wise measures of cortical thickness from it. It should be stressed that neither the two-stage approach nor our approach relies on a specific dataset or a specific type of data. We focus on these two specific datasets because these images are easier to access and do not require any further preprocessing, which provides a fairer comparison set-up between methods. The 3D image resolutions are (96 × 112 × 96) for the Cam-CAN dataset and (141 × 120 × 178) for the ABIDE dataset. To further compare our approach and the two-stage approach, we manually removed some participants from both datasets to create two more modified datasets. Our approach and the two-stage approach will then be compared on all four datasets. The description of each dataset is provided below and Figure 5 shows their chronological age distributions:

#### • Cam-CAN dataset

The Cam-CAN dataset contains 653 cognitively normal participants (mean age 54.3 years, standard deviation 18.5 years, range 18-88 years).

#### • Skewed Cam-CAN dataset

The Cam-CAN dataset has a roughly balanced age distribution with slightly more elderly individuals. We removed 70% of the participants with ages smaller than 40 years and 50% of the participants with ages ranging from 40 years to 60 years to create a skewed age distribution. The skewed Cam-CAN dataset contains 423 cognitively normal participants (mean age 62.2 years, standard deviation 16.9 years, range 18-88 years).

#### • ABIDE dataset

The ABIDE dataset contains 571 cognitively normal participants (mean age 17.1 years, standard deviation 7.7 years, range 6-56 years).

#### • Symmetric ABIDE dataset

The ABIDE dataset has a highly skewed age distribution toward young ages. We removed the participants whose age is larger than 20 years to make this dataset have a more symmetric age distribution.

The symmetric ABIDE dataset contains 415 cognitively normal individuals (mean age 13.2 years, standard deviation 3.1 years, range 6-20 years).

### B Models

To validate that the skewed loss is robust to different network architectures, we implemented three networks roughly based on ResNet [14], VGG [15], and GoogleNet [28] architectures. We replaced the 2D convolution layers with 3D convolution layers [29] and the specific implementations could be found in the GitHub repository mentioned in Section I. As for the choices of model-dependent parameters, such as the number of filters in each layer, we followed a conventional design strategy [30]. All models contain several repeated blocks, each of which contains convolutional layers, activation functions, and batch normalization [31]. The number of filters was set to eight in the first block and was doubled after each max-pooling layer to infer a rich representation of the brain. It should be also noted that different choices of the model-dependent parameters do not affect the final ADC value as neither correction approach focuses on the model architectures. We denote our models as ResNet, VGG, and GoogleNet respectively in the rest of this paper. Compared with the original network architectures, our models have fewer layers due to the size of the dataset and the number of parameters is reduced below 1 million. All models only take the raw images as input and demographic variables such as gender are not included to follow the conventions of brain age prediction problem [8], [9].

Also, we would like to stress that the brain age prediction model is a regression model and hence the bias always exists regardless of different model architectures. The skewed loss and the two-stage approach can both be applied to any model architectures and we only select ResNet, VGG and GoogleNet architecture because they are still widely used in practice [2], [8], [17], [18] and form the building blocks for more complex models.

### C Training and Testing

The skewed loss approach is generic and can be applied to any symmetric loss function. However, in the brain age prediction literature, MAE (i.e. average L1 loss) is the most commonly used metric. Therefore, we focus our analyses on L1 loss.

Because the size of the datasets we used is relatively small, to prevent models from over-fitting, we applied data augmentation in the training process using TorchIO [32]. In each training iteration, every input image had a probability of 50% being flipped around the horizontal plane. Also, the L2 weight decay coefficient was set to 0.001.

During the training process, the Adam optimizer [33] was used as the default optimizer for all models. The initial learning rate was set to 0.01 and then multiplied by 0.5 every 50 epochs. The batch size was set to 16 as default and the total number of epochs was set to 400.

When we trained the model using the skewed loss, the hyperparameters used in Table I are set the same for all models. We set hyperparameter *α* to 150, *β* to 300, *ε* to 5, *θ* to 0.15, and *η* to 1.5.

Datasets were split using a stratified split strategy of which 80% was used for training, 10% for validation, and 10% for testing. To make full use of the whole dataset, we applied a split strategy similar to cross-validation. We randomly split each dataset in 20 different ways so that no two train/validation/test sets are identical. Also, to minimize the fluctuations of the model results due to random initialization of the weights, we trained our model on each train/validation/test split 5 times. In that way, we ended up training a specific model on a particular dataset 100 times.

## VI Results

The performance comparisons between the two-stage approach and the skewed loss across four datasets described in Section V-A are listed in the following subsections. We also add the performances using the normal loss function before the correction stage as comparisons. The ADC is measured using Pearson’s r unless specified otherwise. In Section VI-C, we provide the performance using Spearman’s rank correlation coefficient to measure ADC to illustrate the flexibility of our approach.

Pearson’s r measures statistical dependence between two sets of data in terms of the linear correlation of the variables. Spearman’s rank correlation measures linear correlation between the rank values of two variables and hence quantifies monotonic relationships [34]. The range of both correlation coefficients lies between -1 (negative correlation) and 1 (positive correlation).

It should also be noted that, unlike normal loss functions, correction approaches try to sacrifice model accuracy for more unbiased model predictions. Thus, all correction approaches will result in an increase in MAE compared with normal loss. In our experiments, model performance using normal loss could be regarded as the lower bound of model errors.

To save space, Table II lists some notations used in the following subsections.

### A Model Performance

From Section V-C, we split each dataset using 20 different ways and for each split, we train the network 5 times. At the end of the training, we calculate the average value of the MAE and ADC from the 5 repeated runs to reduce fluctuations in model performances. In that way, we end up with 20 pairs (one split for one pair) of averaged MAE and average ADC between the two-stage approach and skewed loss. The significance tests are performed by comparing the averaged MAE and ADC values across the 20 splits. The model performance across four datasets is shown in Table III and the significance test results between the two-stage approach and the skewed loss are shown in Table IV.

From Table III, the skewed loss achieves a lower MAE compared with the two-stage approach for most models. The averaged ADC of the skewed loss almost always falls within −0.1 to +0.1, which indicates the bias in the brain-age delta has been significantly reduced compared with normal loss. On average, the skewed loss increases the MAE by 0.2 years for the Cam-CAN datasets and 0.97 years for the ABIDE datasets, whereas the two-stage approach increases the MAE by 0.28 years and 1.12 years.

From Table IV, some models using the skewed loss achieve significantly lower MAE compared with the two-stage approach, for example, GoogleNet on Cam-CAN and symmetric ABIDE datasets; ResNet on symmetric ABIDE dataset and VGG on Cam-CAN dataset. For the rest models, the skewed loss is likely to achieve lower MAE compared with the two-stage approach whereas the improvement is not significant enough.

To sum up, both the two-stage approach and the skewed loss correct the model to have a near-zero ADC at the cost of an increase on MAE, whereas using the skewed loss achieves comparable or significantly lower MAE compared with the two-stage approach.

### B Model Performance of Ensemble Models

In Section VI-A, we calculate the MAE of each model and take the average of MAE from the 5 repeated runs. Alternatively, we can create an ensemble model by averaging the model predictions of 5 repeated runs and then calculate its MAE and ADC. Because we split the dataset in 20 different ways, we also end up with 20 ensemble models and thus 20 pairs of MAE and ADC for each model architecture. The ensemble model performance across four datasets is shown in Table V and the significance test results between the two-stage approach and the skewed loss are shown in Table VI.

From Table V, the skewed loss also achieves a lower MAE compared with the two-stage approach for most models. The ADC of ensemble model using the skewed loss almost always falls within -0.1 to +0.1, which also indicates the bias has been significantly reduced compared with normal loss. On average, the skewed loss increases the MAE by 0.08 years for the Cam-CAN datasets and 0.6 years for the ABIDE datasets, whereas the two-stage approach increases the MAE by 0.12 years and 0.78 years.

From Table VI, ResNet on ABIDE dataset, VGG on ABIDE dataset, and GoogleNet on ABIDE and symmetric ABIDE datasets achieve significantly lower MAE using the skewed loss compared with the two-stage approach. For the rest models, the skewed loss tends to achieve lower MAE compared with the two-stage approach whereas the improvement is not significant.

To sum up, for ensemble models, the skewed loss can achieve comparable or significantly better performances compared with the two-stage approach. Using the skewed loss in ensemble models can also remove the bias in the brain age delta.

### C Performances Using Spearman’s Rank Correlation as ADC

**Fig. 6:**
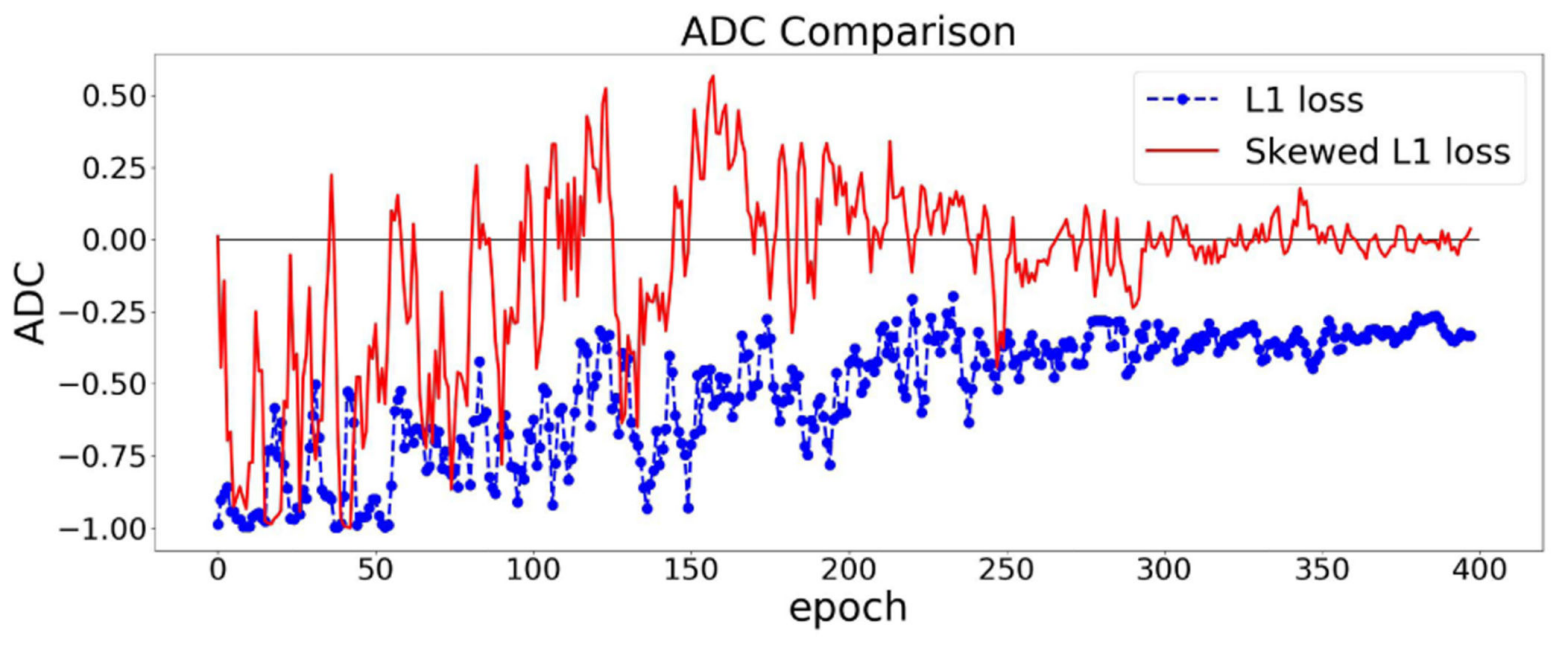
ADC Comparisons between L1 and skewed L1 loss. Dynamic lambda strategy is applied for the skewed loss. The model is trained twice using L1 loss and skewed L1 loss respectively. The dashed and solid lines represent the changes of ADC (measured in Spearman’s rank correlation) on the validation set in the training process using L1 and skewed L1 loss respectively.

A further experiment is carried out to evaluate how our approach performs by changing the ADC from Pearson’s r to other correlation metrics. Here, due to space constraints, we only showed the performances using ResNet on the Cam-CAN dataset with ADC measured in Spearman’s rank correlation in Table VII and Figure 6.

In Figure 6, we again train our model using L1 loss and skewed L1 loss with dynamic lambda strategy. We can observe that switching to different types of correlation metrics results in the specified metric approaching zero. It shows the potential to be generalized to other types of correlation metrics.

### D ConsistencyofCorrelation Trends

Figure 3, Figure 4, and Figure 6 show the changes of correlation of a single model. To further validate the stability of our approach, we can examine how the variance of ADC changes in the training process. A large variance of ADC indicates large oscillations in ADC and vice versa.

**Fig. 7:**
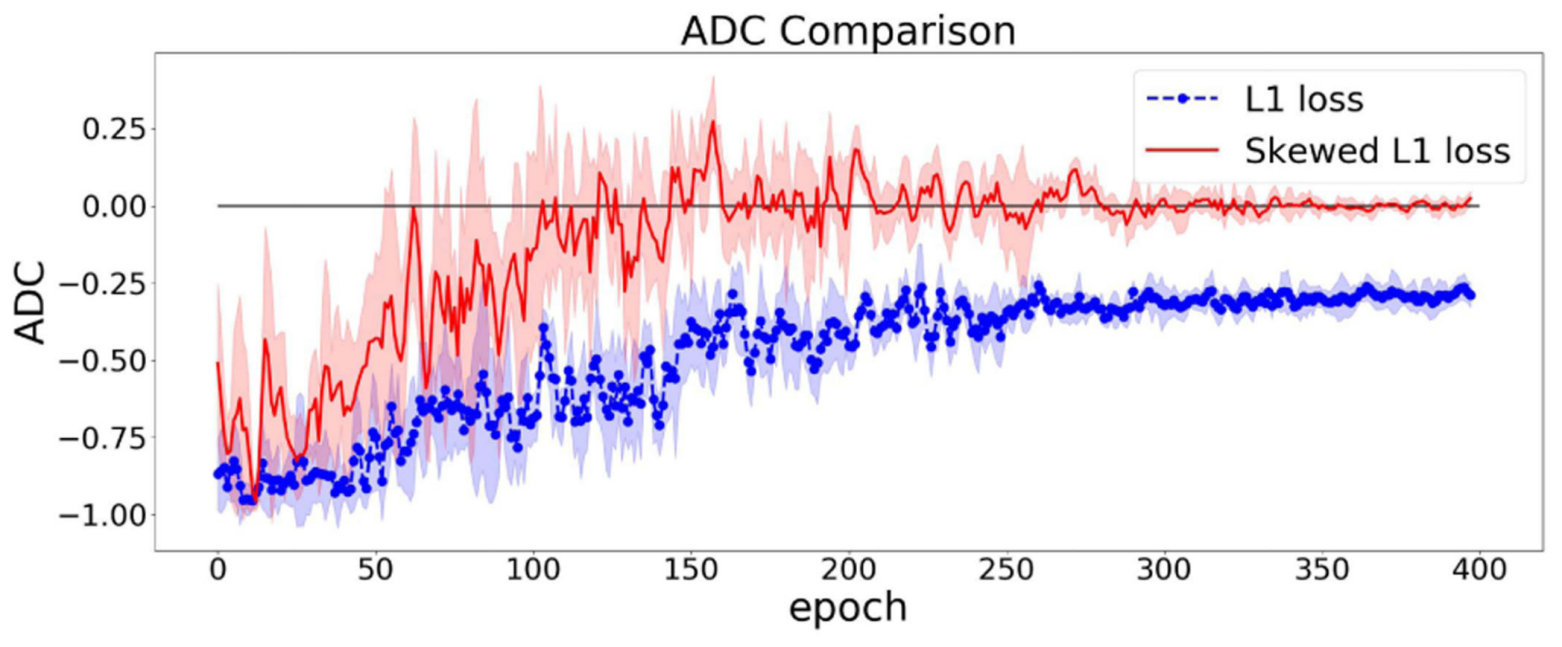
Averaged ADC Comparisons between L1 and skewed L1 loss. The dashed and solid lines represent the changes of ADC on the validation set in the training process using L1 and skewed L1 loss respectively. The shaded area represents the mean ADC plus or minus one standard deviation.

In Section V-C, we train each model 5 times to reduce the fluctuations. Thus, we group these 5 runs to calculate the mean and standard deviation of ADC. Figure 7 shows the trend of averaged ADC of the 5 repeated runs.

From Figure 7, we can observe that the variance of ADC decreases in the training process and arrives at a small value at the end of training. This indicates the ADC can almost always arrive at (near) zero using the skewed loss on the validation set, which further validates the stability of the skewed loss.

### E Robustness to Data Distribution Shift

Machine learning models typically require that the training and test set should share some similar properties like data distributions and the models are expected to learn these similarities during the training process. However, due to the variability of the unseen test data, these similarities may not always hold, which inevitably causes performance degradation in most cases.

Although learning with distribution shift has been extensively studied in recent years, it remains a challenging and ongoing topic in machine learning. In Section VI-A, we have discussed the model performance of the skewed loss without distribution shift. Therefore, it is crucial to investigate whether the skewed loss is robust to distribution shift or not.

#### 1) Experiment Settings

To make the test set significantly different from the training set, we manually split our dataset based on the participants’ chronological age. For illustration purposes, we only selected the Cam-CAN dataset and ResNet architecture to discuss the distribution shift problem. The Cam-CAN dataset was split into three groups. Group 1 consists of participants with ages below 40 years. Group 2 consists of participants with ages above 40 years and below 60 years. Group 3 consists of participants with ages above 60 years.

**Fig. 8:**
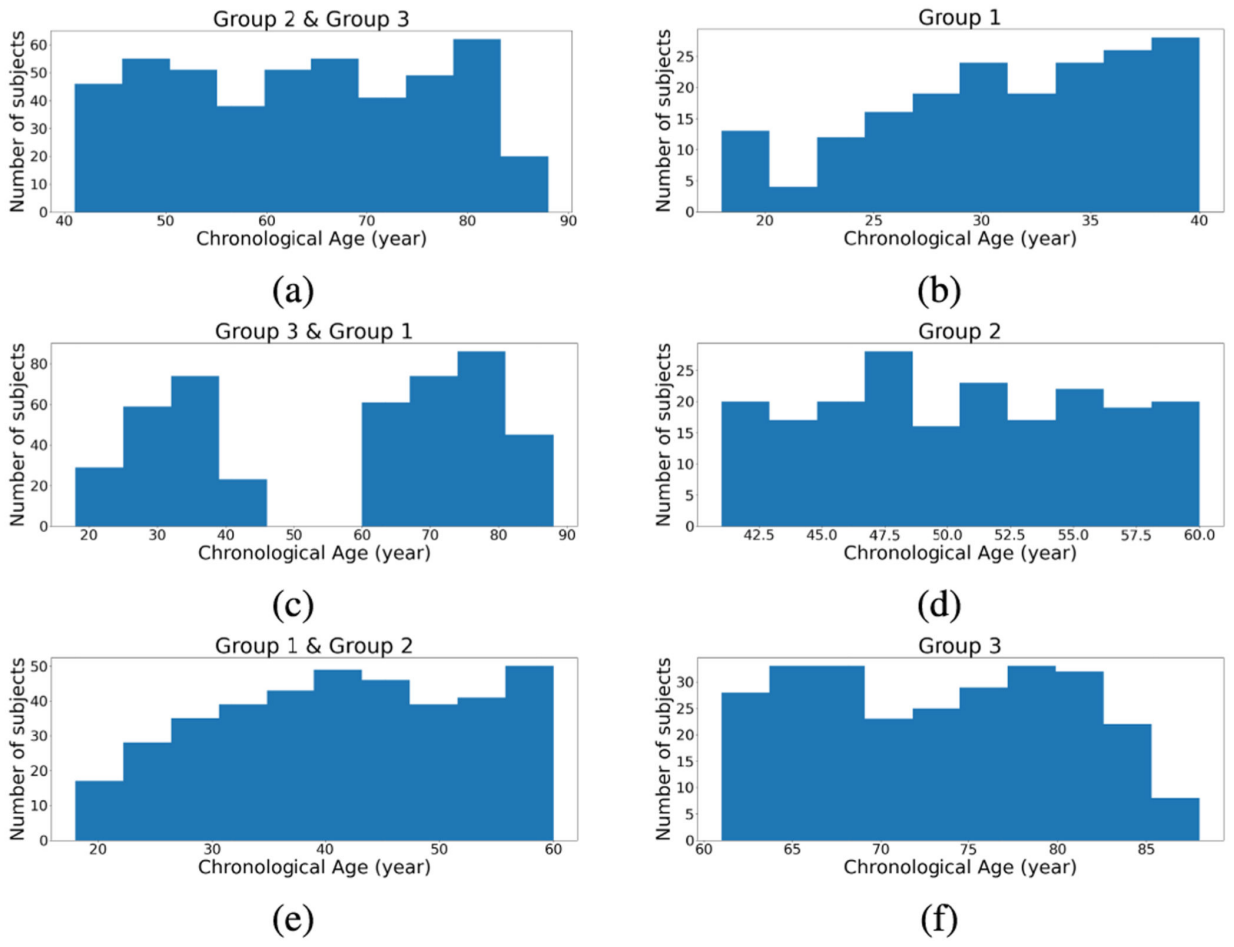
Age distributions of three types of train-test split. Each row represents a different way of creating the training and test set. The left three plots represent the three training sets and the right ones represent the corresponding test sets.

The model was then trained three times using each correction approach. For each time, one group was selected as the test set and the remaining two groups were combined as the training set. The chronological age distribution of the training and test sets of the three train-test splits are illustrated in Figure 8.

#### 2) Results

The model performance comparisons on three different train-test splits are shown in Table VIII. The correlation is measured in Pearson’s r.

#### 3) Discussions

From Table VIII, it could be observed that neither the skewed loss nor the two-stage approach achieves a near-zero ADC at the end of the training, which indicates that neither approach works well when facing distribution shifts.

However, by comparing the skewed loss and the two-stage approach, it could be observed that the skewed loss suffers less in terms of both MAE and ADC in all three experiments. Moreover, when Group 1 or Group 3 is selected as the test set, both correction approaches decrease the MAE compared with the normal loss, which is not observed in Table III and Table V.

The reason why the skewed loss and the two-stage approach are not robust to distribution shift is that they both heavily rely on the validation set. The two-stage approach relies on the validation set to calculate *β*_0_ and *β*_1_ in (5), whereas the skewed loss aims to achieve a near-zero ADC on the validation set. For both approaches, the validation set is used as the target for the model to optimize. If the validation set is significantly different from the test set, both methods will suffer.

There is one more point to be noted by comparing Table VIII and Table III, it could also be observed that MAE increases significantly. There are several reasons for it. Firstly, the size of the training set becomes much smaller compared with the one used in Section V. Secondly, take the first train-test split (Group 1 as the test set) as an example. In the first train-test split, the training set only contains subjects whose age is above 40 years so that the model is also more likely to give a prediction that is above 40 years, whereas the true labels from Group 1 are all below 40 years. Therefore, when using Group 1 and Group 3 as the test set, the MAE increases significantly.

To sum up, although both the two-stage approach and the skewed loss are not robust to distribution shift, the skewed loss suffers less compared with the two-stage approach in terms of both MAE and ADC.

## VII Generalization of the Skewed Loss

We have demonstrated the ability of the skewed loss in removing the dependence of brain age delta on chronological age. We would like to stress that the skewed loss could also be generalized to other areas regardless of brain age prediction. We provide a simple example here to illustrate the generalization of the skewed loss using the apparent age prediction problem. It should also be noted that in apparent age prediction, achieving a more accurate prediction (a lower MAE) is the primary goal rather than a near-zero correlation as in brain age prediction.

### A ApparentAgePredictionExplained

Predicting a person’s real age based on a single face image is a classic problem in the computer vision field. However, the model performance can often be affected by outliers represented by people who have an appearance that is not in line with their real age [35]. Therefore, a different approach has been developed known as apparent age prediction, which is the age perceived by humans. The problem can then be formulated as building a regression model that takes a person’s face image as input and uses the apparent age as output.

### B Experiments

To empirically investigate our approach on apparent age prediction, we conducted experiments using a publicly available face dataset. The models were trained using the normal symmetric loss and the skewed loss respectively. The two-stage approach was also applied to models using the normal symmetric loss as the contrast.

#### 1) Dataset

We selected the ChaLearn Looking at People 2015 competition dataset (LAP dataset), which provided thousands of annotated images [36], [37]. The images were labeled based on web applications that averaged the opinion of 10 independent users to obtain the apparent age.

To preprocess the original dataset, we first ran a face detection program on all images to remove the useless background information. The program could be found in a GitHub repos-itory^4^ and we applied the default HOG-based approach [38] to extract the face from the whole image. The resulting face images were then resized to have a spatial resolution of 100 × 100 pixels. In total, we obtained 4383 face images.

#### 2) Models

We adopted the ResNet [14] architecture to demonstrate the effectiveness of the skewed loss in this problem. As for the choices of model-dependent hyperparameters, we also followed the conventional design strategy discussed in Section V-B. The specific implementations could also be found in the GitHub repository mentioned in Section I.

#### 3) Training and Testing

The general training and testing strategy stays almost the same as the brain age prediction. Because of the size of the LAP datasets, to prevent models from over-fitting, we applied data augmentation in the training process. In each training iteration, every input image had a probability of 50% being flipped horizontally. Also, the L2 weight decay coefficient was set to 0.001.

During the training process, the Adam optimizer [33] was used as the default optimizer for all models. The initial learning rate was set to 0.01 and then multiplied by 0.5 every 50 epochs. The batch size was set to 64 as default and the total number of epochs was set to 300.

When we trained the model using the skewed loss, the hyperparameters used in Table I are set the same for all models. We set hyperparameter *a* to 50, *β* to 150, *e* to 3, *θ* to 0.15, and *η* to 1.2.

Datasets were split using a stratified split strategy of which 80% was used for training, 10% for validation, and 10% for testing. Also, to minimize the fluctuations of the model results due to the random initialization of the weights, we trained our models using skewed loss and normal loss 5 times respectively.

### C Results

**Fig. 9:**
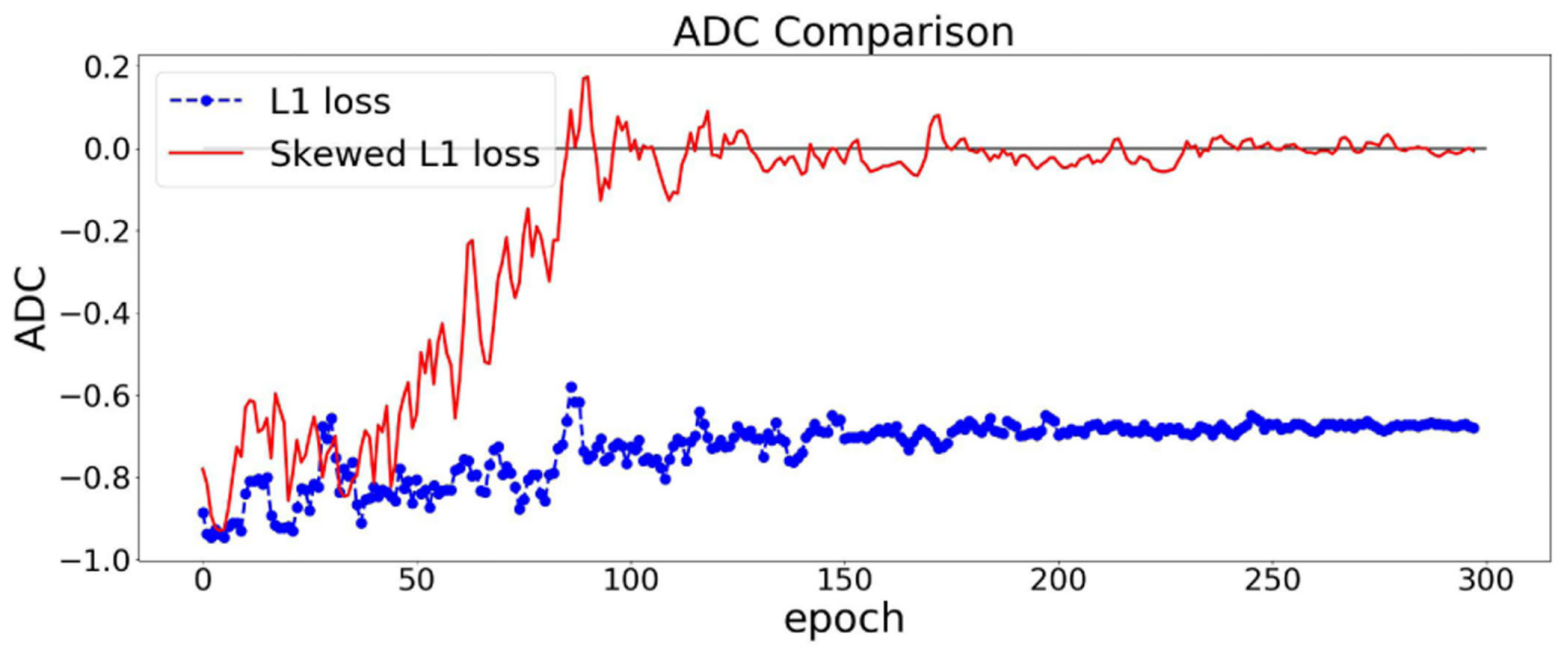
ADC Comparisons between L1 and skewed L1 loss. Dynamic lambda strategy is applied for the skewed loss. The model is trained twice using L1 loss and skewed L1 loss respectively. The dashed and solid lines represent the changes of ADC (measured in Pearson’s r) on the validation set in the training process using L1 and skewed L1 loss respectively.

The model performance comparisons on apparent age prediction are shown in Table IX and Figure 9. The correlation is measured in Pearson’s r.

It should be noted that considering the small size of this dataset and low spatial resolution (100 × 100), the MAE should still be considered acceptable compared with other studies [35], [39].

From Figure 9, We can observe that the skewed loss also achieves a near-zero correlation at the end of the training, which proves that the skewed loss could be generalized to other areas regardless of the brain age prediction.

## VIII Discussion

We have proposed a skewed loss function and dynamic lambda training strategy to solve the nonzero ADC problem in brain age prediction. The skewed loss counteracts this bias by switching the normal symmetric loss function into a skewed form. The dynamic lambda strategy tunes *λ_max_* iteratively to search for the optimal value that can enable the model predictions to have a near-zero ADC at the end of training.

One assumption of our approach is that models with zero ADC on the validation set should also have near zero ADC on the test set. By evaluating model performances in Section VI, we find that using the skewed loss, ADC on the test set always has a mean around zero, which supports our assumption.

The most significant difference between the skewed loss and the two-stage approach is that the skewed loss controls ADC explicitly whereas the two-stage approach controls it implicitly. That means changing the way we measure ADC from Pearson’s r to other types of correlation metrics, our approach could have a similar effect with the specified correlation metric approaching zero. In Section VI-C, we showed that using Spearman’s rank correlation can also achieve similar performances.

We also demonstrated in Section VI that both the skewed loss and the two-stage approach can achieve a near-zero ADC, whereas the skewed loss achieves comparable or even better model performances in terms of MAE compared with the two-stage approach. Theoretically speaking, a two-stage approach leads to a result that is strictly worse in terms of minimizing both MAE and ADC than a single integrated approach. In the two-stage approach, the model is optimized to minimize MAE. When correction is applied afterward, there is no guarantee that the MAE remains low. In an integrated approach, the model is optimized to minimize MAE conditioned on a desired maximum value of ADC [13]. Knowledge of the constraint allows the model to find a better local minimum to solve the task of minimizing both MAE and ADC. Also, the skewed loss only aims to encourage the model to make predictions toward the opposite side, i.e., from overestimations toward underestimations for young individuals and vice versa. In terms of MAE, there is no difference between overestimations and underestimations. In Section VI-E, we also evaluated whether the correction approaches are robust to distribution shifts. Although neither approach always achieves a near zero ADC, the skewed loss has been proved to suffer less in terms of both MAE and ADC from Table VIII.

We have also demonstrated in Section VII that the skewed loss, as well as the two-stage approach, could also be generalized to other areas. The observed bias is also commonly known as the regression dilution bias, which could be found in almost any regression model. However, whether to apply the skewed loss, as well as other approaches, depends on different applications. In brain age prediction, a near-zero ADC is the primary goal as a non-zero ADC could cause spurious relationships in subsequent experiments, whereas in apparent age prediction, a more accurate age estimate is more important.

It should also be noted that both the two-stage approach and the skewed loss have their advantages and shortcomings. Regarding the two-stage approach, it is easier to implement, because, in each stage, only one metric (MAE or ADC) is optimized. However, when the correction is applied afterward, there is no guarantee that the MAE from the first stage remains low, which can be observed in Table III. As for the skewed loss, it is obvious that by optimizing the two metrics (MAE and ADC) in a unified approach, the model takes care of both metrics at the same time. As for the shortcomings, a successful search of *λ_max_* is crucial for the skewed loss approach. In Algorithm 1, we provide a heuristic way to search for it which proves to be robust. However, the two-stage approach does not require this additional hyperparameter searching.

There are still some aspects that require further improvements. Firstly, it would be preferred to apply the skewed loss on a larger dataset. Secondly, our approach can only be applied in regression models. Peng et al. [8] treated brain age prediction as a classification task and applied a weighted sum to calculate the predicted brain age. The two-stage approach can be applied to that situation while ours cannot. Also, it is useful to apply the skewed loss function in a case study to assess its sensitivity to detect pathological changes in patients. Although our approach achieves a near-zero ADC value that is similar to the two-stage approach, a case study can further confirm the relevance of our approach rather than through the ADC value.

To conclude, we developed the skewed loss function to counteract the predictive bias in brain age prediction. In most cases, it achieves a better performance than the existing two-stage approach. Also, our approach has been verified in different datasets using different neural network architectures. By taking ADC explicitly into consideration in the training process, our approach also shows the potential to remove nonlinear relationships by measuring ADC using relevant correlation metrics.

## Figures and Tables

**Table I:** Notations for Dynami c Lambda Strategy

Notation	Type	Description	Range
γ	hyperparameter	total number of epochs	
*α*	hyperparameter	stage-2 start epoch	γ/3
*β*	hyperparameter	stage-3 start epoch	2γ/3
ε	hyperparameter	update interval of λ_wαa;_	[5, 10]
*ADC*	variable	age-delta correlation	
M	variable	an array to save *λ_max_*	
N	variable	an array to save *ADC*	
*θ*	hyperparameter	correlation threshold	**[0.1, 0.2]**
*η*	hyperparameter	a positive multiplier	**(1.2)**
${ℒ}_{s}(\lambda_{max})$	function	skewed loss controlled by *λ_max_*	

**Table II:** Notations for Performance Comparisons

Notations	Descriptions
Normal loss	models using normal loss without two-stage approach
Two-stage	models using normal loss with two-stage approach
Skewed loss	models using the skewed loss with dynamic lambda strategy
Wilcoxon	Wilcoxon signed rank test
Paired-t	paired t-test

**Table III:** Model Performances on Four Datasets

Dataset	Model	Normal loss		Two-stage		Skewed loss	
		MAE	ADC	MAE	ADC	MAE	ADC
Cam-CAN	ResNet	4.80±0.42	-0.28±0.I2	5.06±0.49	-0.03±0.I4	**4.96+0.50**	-0.03±0.I3
	VGG	5.09+0.44	-0.23+0.11	5.35+0.56	-0.02+0.14	**5.22±0.59**	-0.05+0.12
	GoogleNet	5.10+0.54	-0.25+0.13	5.44+0.68	-0.03+0.14	**5.22±0.60**	-0.05+0.15
Skewed Cam-CAN	ResNet	5.02±0.70	-0.35+0.19	5.3l±O.7l	-0.04±0.20	**502l±0.76**	-0.07±0.20
	VGG	5.23+0.66	-0.34+0.17	**5,42±0.7l**	-0.06+0.19	5.43+0.75	-0.06+0.17
	GoogleNet	5.28±0.7l	-0.38+0.15	**5.62±0.79**	-0.05±0.I9	5.67±0.76	-0.06±0.I8
ABIDE	ResNet	3.40±0.43	-0.62+0.15	4.7l±0.97	-0.04±0.28	**4.54±0.66**	-0.08±0.26
	VGG	3.33+0.32	-0.6+0.15	4.35+0.89	-0.06+0.24	**4.I9±0.69**	-0.09+0.25
	GoogleNet	3.37±0.37	-0.60+0.15	4.50±0.75	-0.08±0.25	**4.36+0.65**	-0.I2±0.27
Symmetric ABIDE	ResNet	2.08+0.27	-0.73+0.08	3.55+0.88	-0.05+0.20	**3.28±0.81**	-0.09+0.23
	VGG	1.92+0.26	-0.64+0.11	2.81+0.60	-0.07+0.21	**2.8l±0.57**	-0.03+0.24
	GoogleNet	2.03±0.53	-0.64±0.ll	2.93±0.73	-0.05±0.24	**2.76+0.61**	-0.07±0.23

**Table IV:** Significance Test Between Two-Stage and Skewed Loss With p-Values

Dataset	Model	Wilcoxon		Paired-t	
		MAE	ADC	MAE	ADC
Cam-CAN	ResNet	0.09	0.77	0.06	0.76
	VGG	**0.06**	0.16	**0.06**	0.21
	GoogleNet	**<0.01**	0.19	**<0.01**	0.12
Skewed Cam-CAN	ResNet	0.10	0.14	0.28	0.14
	VGG	0.50	0.91	0.85	0.98
	GoogleNet	0.85	0.91	0.72	0.66
ABIDE	ResNet	0.70	<0.01	0.19	<0.01
	VGG	0.09	0.16	0.12	0.05
	GoogleNet	0.18	0.19	0.13	0.17
Symmetric ABIDE	ResNet	**<0.01**	0.07	**<0.01**	0.05
	VGG	0.82	0.10	0.99	0.09
	GoogleNet	**0.01**	0.45	**<0.01**	0.41

**Table V:** Ensemble Model Performances on Four Datasets

Dataset	Model	Normal loss		Two-stage		Skewed loss	
		MAE	ADC	MAE	ADC	MAE	ADC
Cam-CAN	ResNet	4.00±0.24	-0.34±0.ll	4.I2±0.29	-0.03±0.I2	**4.07+0.33**	-0.04±0.I2
	VGG	4.12+0.31	-0.29+0.10	4.26+0.39	-0.03+0.11	**4.21+0.36**	-0.06+0.11
	GoogleNet	4.06±0.32	-0.32±0.I3	4.20±0.48	-0.03±0.I4	**4.09±0035**	-0.05±0.I4
Skewed Cam-CAN	ResNet	4.33±0.6l	-0.4±0.I5	4.49±0.57	-0.04±0.2l	**4.38+0.66**	-0.07±0.20
	VGG	4.45±0.57	-0.39±0.I5	4.50±0.56	-0.06±0.I9	**4.45+0.58**	-0.07±0.I7
	GoogleNet	4.30+0.49	-0.46+0.12	**4.38+0.48**	-0.06+0.19	4.55+0.48	-0.07+0.17
ABIDE	ResNet	3.02+0.35	-0.67+0.11	4.04+0.79	-0.04+0.30	**3.66±0.43**	-0.08+0.27
	VGG	3.00±0.25	-0.64±0.I3	3.79±0.76	-0.06±0.25	**3.57+0.51**	-0.09±0.25
	GoogleNet	3.00+0.34	-0.66+0.12	3.89+0.60	-0.08+0.25	**3.65+0.47**	-0.12+0.27
Symmetric ABIDE	ResNet	1.85+0.19	-0.82+0.04	2.67+0.57	-0.05+0.20	**2.52+0.42**	-0.09+0.24
	VGG	I.67±0.I9	-0.73±0.06	**2.20±0.36**	-0.07±0.2l	2.30±0.39	-0.03±0.27
	GoogleNet	l.79±0.50	-0.72±0.07	2.39±0.57	-0.05±0.26	**2.20+0.47**	-0.08±0.24

**Table VI:** Ensemble Significance Test Between Two-Stage and Skewed Loss With p-Values

Dataset	Model	Wilcoxon		Paired-t	
		MAE	ADC	MAE	ADC
Cam-CAN	ResNet	0.19	0.68	0.31	0.73
	VGG	0.46	0.18	0.44	0.20
	GoogleNet	0.14	0.16	0.10	0.13
Skewed Cam-CAN	ResNet	0.28	0.13	0.31	0.13
	VGG	0.41	0.91	0.49	0.78
	GoogleNet	0.14	0.97	0.09	0.65
ABIDE	ResNet	**<0.01**	0.02	**<0.01**	0.02
	VGG	**0.04**	0.26	**0.05**	0.09
	GoogleNet	**<0.01**	0.30	**0.01**	0.28
Symmetric ABIDE	ResNet	0.15	0.17	0.07	0.24
	VGG	0.26	0.18	0.24	0.17
	GoogleNet	**<0.01**	0.39	**<0.01**	0.40

**Table VII:** ResNet Performance on Cam-CAN Using Spearman’s Rank Correlation as ADC

Model	Metric	Normal loss	Two-stage	Skewed loss
ResNet	MAE	4.79±0.55	5.08±0.60	**4.95±0056**
	ADC	-0.33±0.I8	-0.02±0.20	-0.05±0.20

**Table VIII:** Model Performance Comparisons When Facing Distribution Shift

Training Set	Test Set	Metric	Normal loss	Two-stage	Skewed loss
Group 2 & Group 3	Group 1	MAE	15.4+1.51	14.54+1.22	**14.50±1.30**
		ADC	-0.85+0.05	-0.80±0.03	**-0.75±0.03**
Group 3 & Group 1	Group 2	MAE	6.9+0.31	7.75+0.25	**7.44±0039**
		ADC	0.08+0.005	0.14+0.01	**0.11 ±0.01**
Group 1 & Group 2	Group 3	MAE	I5.45±l.l2	ll.4±0.38	**l0.63±l088**
		ADC	-0.7+0.03	-0.6+0.06	**-0.52±0.06**

**Table IX:** ResNet Performance on Apparent Age Prediction

Model	Metric	Normal loss	Two-stage	Skewed loss
ResNet	MAE	8.32±0.22	l5.42±l.22	**I3.64±0.99**
	correlation	-0.75+0.06	0.02+0.04	0.0l±0.06

## Footnotes

1
https://github.com/hanzhiwangchn/MRI_Age_Prediction

2
https://www.cam-can.org

3
http://preprocessed-connectomes-project.org/abide/download.html

4
https://github.com/ageitgey/face_recognition
